# Editorial: Microbial interactions with metals/minerals: from environmental aspects to applications

**DOI:** 10.3389/fmicb.2026.1908894

**Published:** 2026-07-17

**Authors:** Ji-Hoon Lee, Cuong Tu Ho, Dongfei Han, Zeinah Elhaj Baddar, Roberta Amendola

**Affiliations:** 1 Department of Bioenvironmental Chemistry, Jeonbuk National University, Jeonju, Republic of Korea; 2 Institute of Science and Technology for Energy and Environment, Vietnam Academy of Science and Technology, Hanoi, Vietnam; 3 School of Environmental Science and Engineering, Suzhou University of Science and Technology, Suzhou, China; 4 University of Georgia Savannah River Ecology Laboratory, Aiken, SC, United States; 5 Mechanical & Industrial Engineering Department, Montana State University, Bozeman, MT, United States

**Keywords:** biogeochemical cycling, bioleaching, biomining, bioremediation, heavy metal resistance, microbial interactions

Microbial interactions with metals and minerals shape the Earth's biosphere, driving global biogeochemical cycles, weathering rocks, and dictating the mobility of both nutrients and toxic elements. This Research Topic, entitled “*Microbial Interactions with Metals/Minerals: From Environmental Aspects to Applications*,” brings together 17 diverse articles that explore the complex molecular, cellular, and ecological pathways mediating these relationships. From deep-sea volcanic ash layers to the frontiers of space biomining, these contributions highlight how understanding plant–soil–microbe–mineral interactions can yield innovative solutions for environmental sustainability, resource recovery, and pollution mitigation.

## 1 Biogeochemical cycling of metals and minerals

Several studies in this Research Topic expand our understanding of how microbially mediated redox reactions alter geological substrates under diverse environmental conditions. Moving into deep biosphere regimes, Rolfes et al. show that ancient basaltic tephra (volcanic ash) layers in marine sediments harbor unique microbial communities capable of exploiting specific “reducible” Fe(III) oxides, suggesting how life flourishes in carbon-lean sub-seafloor environments. On the surface, the dissolution kinetics of such substrates are further analyzed by Yunda et al., who show that the hyperthermophilic archaeon *Pyrobaculum islandicum* enhances the dissolution of Fe(III)-rich basaltic glass through active Fe(III) reduction.

In freshwater systems, seasonal transitions profoundly dictate metal mobility. Hahn et al. explore how thermal stratification and subsequent anoxia drive prokaryotic community shifts that accelerate manganese (Mn^2+^) and iron release from reservoir sediments. The physiological mechanisms of these iron-reducing pathways are elegantly tied to environmental detoxification by Takahashi et al., who reveal that bromate reduction by *Shewanella* species depends on an interconnected system of endogenous *c*-type cytochromes and exogenous iron mediators that chemically reduce carcinogenic bromate into harmless bromide. Furthermore, the physical constraints of mineral matrices on microbial enzymes are underscored by Ai et al., who show how coal matrix structures directly govern the organic desulfurization efficiency of *Pseudomonas putida* via the 4S metabolic pathway.

## 2 Mercury biogeochemistry and speciation

Mercury (Hg) pollution remains a severe global threat due to the microbial synthesis of neurotoxic methylmercury (MeHg). This Research Topic also provides interesting insights into Hg transformations across different spatial scales. At the sub-cellular level, Meng et al. utilize Hg L_*III*_-edge EXAFS to map the compartment-resolved distribution of thiol functional groups in *Geobacter sulfurreducens*, revealing how exceptional thiol densities within the cellular membranes govern the retention, internalization, and ultimate methylation of soft metals.

At an ecosystem scale, Tada et al. simultaneously map the functional genes responsible for Hg methylation (*hgc*AB), demethylation (*mer*B), and reduction (*mer*A) within the western North Pacific Subtropical Gyre, identifying *Nitrospina* lineages as dominant open-ocean methylators whose activities are linked to nitrogen cycling. On land, Yu et al. track the ecological fallout of long-term mercury mining in China, detailing how agricultural soil bacterial communities undergo adaptive restructuring to maintain metabolic potential despite severe Hg stress, pointing to key metal-resistant biomarker genera (*Rokubacteriales, Gaiella*) that could assist future restoration projects.

## 3 Bioremediation of agricultural and industrial sites

The co-contamination of soils with heavy metals like cadmium (Cd) and lead (Pb) threatens food security and public health. Articles in this Research Topic offer novel, multi-step strategies to mitigate these risks. Chen et al. present a highly effective synergistic remediation method for paddy soils, combining iron-based sulfur-rich material with foliar zinc fertilizers to immobilize Cd/Pb, enrich iron/sulfur/nitrogen-cycling taxa (*Geobacterales, Desulfobacterota*), and drastically reduce heavy metal accumulation in rice grains. In a similar vein, Gao et al. introduce the paradigm of nutrient spatial heterogeneity, proving that non-uniform nutrient distribution enhances the compensation capacity and hormetic tolerance of soil respiration under Cd stress.

At the organismal level, Wang et al. decrypt the genetic toolkit of the filamentous fungus *Paecilomyces lilacinus*, identifying CreA, ZnT, and MTase as pivotal regulators governing Cd resistance, where CreA was identified as a “master regulator,” thereby providing clear targets for genetic engineering in bioremediation. Finally, looking at real-world ecological scars, Yan et al. investigate how iron ore mining operations disturb plant-microbe alliances, showing how distinct native flora actively enrich specific multidrug-resistant or oligotrophic rhizosphere bacteria to withstand severe nutrient deficiencies and heavy metal stress.

## 4 Biomining, bioleaching, and sequestration

Harnessing microbes to extract valuable or critical elements offers a sustainable alternative to conventional, chemically intensive mining. This Research Topic is evaluated by Bhatti and Tuovinen, who review the mechanisms of heterotrophic bacterial bioleaching and cellular sequestration of uranium, emphasizing the roles of organic acids and extracellular polymeric substances (EPS) in shifting uranyl ions into bio-unavailable phases. Complimenting this, Yang et al. isolate an indigenous *Shewanella putrefaciens* strain from radioactive tailings that achieves a 93% uranium removal efficiency. Yang et al. also demonstrated the function of –NH_2_, –COOH, and phosphate groups in the biosorption of uranium and utilize genome sequencing and Mtr-pathway knockouts to map out the extracellular electron transfer chain responsible for uranium immobilization.

Expanding the potential of fungal systems, Liu et al. utilize transcriptomics and DNA methylomics to outline how *Aspergillus niger* tolerates and adsorbs the rare earth element cerium(III), revealing a dual-defense mechanism involving extracellular chemical monolayer binding and targeted metabolic rerouting. In addition, the boundaries of biomining are pushed into Earth orbit by Tonietti et al., who assess the space-biomining candidate *Sphingomonas desiccabilis* across terrestrial and extraterrestrial rock types (including meteorites), proving that bioleaching efficiency is highly dependent on substrate mineralogy, which would be an important finding for future *in situ* resource utilization (ISRU) during long-duration space missions. Wrapping up these biotechnological frameworks, Mondal et al. contribute a comprehensive review that frames quorum sensing as a key regulatory dial controlling biofilm dynamics, heavy metal resistance, and metabolic optimization during bioremediation.

## 5 Conclusion and future outlook

Collectively, the articles within this Research Topic show that microbial interactions with metals and minerals are not just isolated chemical reactions. Instead, they are highly dynamic and coordinated processes driven by cellular localization, the surrounding environment, nutrient availability, and community signaling. The insights gathered here not only deepen our fundamental knowledge of terrestrial and marine biogeochemistry but also provide us with versatile biotechnological tools to clean up industrial pollution, secure agricultural health, and sustainably harvest critical resources on Earth and beyond. We extend our sincere gratitude to all the authors, reviewers, and editors who contributed their expertise to make this Research Topic a milestone in the field of environmental microbiology.

